# On the Use of Nitrate of Silver in Vaginal Discharges

**Published:** 1829-10

**Authors:** George Jewel


### VAGINAL DISCHARGES.

### *On the Use of Nitrate of Silver in Vaginal Discharges.*

By George Jewel, Esq.

There are no diseases, to which the female system is liable, more common, or, to a superficial observer, more diversified or anomalous in their character, than those which are attended by vaginal discharges. So intractable, indeed, do they sometimes prove, as to induce, by their long continuance, even under ordinary circumstances, the severest dyspeptic symptoms, feverish paroxysms, hysterical uneasiness, excessive languor, and emaciation; or, by operating upon the brain through the medium of the digestive organs, occasion other sympathetic affections, still more serious in their nature and termination.

It must be familiar to the practitioner, that every discharge which issues from the vagina, not sanguineous, is among females usually included in the term Leucorrhœa, or "whites." There is also a very popular opinion that vaginal discharges have their origin in constitutional or local debility: hence a complaint of this kind is denominated a "weakness." That such a term should be employed to perpetuate an error in practice, is to be lamented; for I believe, if we investigate into the pathology of leucorrhœa, we shall find, for the most part, general or local increased action to be the exciting cause.

It would appear, from a strict investigation into the numerous causes of leucorrhœal complaints which have fallen under my observation, that one uterine affection gives rise to vaginal discharge more frequently than any other, namely, a subacute or chronic inflammation of the cervix uteri. I am disposed to believe, also, that very many of such cases are mistaken for carcinoma uteri, and that, in consequence, either no remedies are prescribed, or a very inefficient mode of practice is adopted. I am aware that, in many cases the train of symptoms about to be noticed may be attributed to an irritable condition of the uterus, so ably described by Dr. Gooch. I cannot, however, easily relinquish the opinion I had originally entertained upon the subject, namely, that inflammation, either of the chronic or subacute kind, of the cervix uteri is, in the majority of cases, the exciting cause of vaginal discharge. The distinction, however, although pathologically recognised, cannot, I conceive, be material in practice: indeed, this will be obvious to the talented author himself, whose mode of practice, in cases of irritable uterus, appears precisely applicable to cases of chronic uterine affections generally. Again, in some cases it may be difficult to discriminate between such diseases as I have alluded to, and incipient scirrhous disorganization. The following remarks will probably assist the young practitioner in his diagnosis.

This inflammation of the cervix uteri, like scirrhus or other organic disease of the uterine system, attacks occasionally at the period of life when the catamenia are about to cease; but I have more frequently found it to exist in married women, from the age of twenty-six or twenty-seven to that of forty, and very recently I have seen several severe cases occurring in young married females, within three months after the birth of the first child. The local symptoms in both diseases are very nearly allied, namely, occasional lancinating pain, more or less acute, through the region of the uterus; with a constant dull kind of pain about the inferior portion of the sacrum, the hip, or groin; attended also by an irritable bladder, or frequent desire to void the urine, and in some severer cases by tenesmus. The vaginal discharge is of a milky or cream-like colour, and is commonly, but particularly in the more acute cases, mixed with a dark-coloured or grumous secretion. Upon making an examination per vaginam in this disease, the os uteri will not be found opened to the same extent as in carcinoma, nor will its margin present the same cartilaginous hardness to the touch. The pain does not appear to be situated in the edges of the os uteri, as described by Mr. Burns, but in the cervix, as pressure upon this part alone occasions the patient to complain. The uterus will be found projecting lower in the vagina than natural; but this will depend upon the nature of the complaint: the more acute, the farther it will have descended.

It is not my intention to dwell upon the routine practice usually had recourse to in uterine diseases; such as the local abstraction of blood, perfect rest, narcotics, the warm bath, &c.; but rather to draw the attention of the profession to a therapeutical agent, which I believe has never, or to a very limited extent, been employed in such cases, namely, the nitrate of silver applied directly to the part affected; a practice which I have been led to adopt from having so frequently witnessed the extensive and healthy changes which have resulted from the application of this remedy to the different mucous tissues, when their secreting surfaces had taken on a disordered or unhealthy action. The mode I have adopted in its application has been either to conceal it in a silver tube, precisely upon the principle as it is employed in cases of stricture, (except that the tube should be adapted to the size of the caustic,) or in the form of solution, in the proportion of three grains to the ounce of water, the strength being gradually increased. A bit of sponge, firmly and neatly tied to a piece of whalebone, is to be moistened with the solution, and carefully introduced into the vagina up to the os and cervix uteri. This mode of application is preferable to the injection, and can easily be effected by the patient herself. The application should be frequently made, or no permanent good can be anticipated.

The following cases, which I have selected from others in consequence of their having been unusually protracted and severe, will exhibit the mode of treatment successfully practised.

I. Feb. 24th.—Mrs. C., ætat. thirty-three, had been delivered, three years ago, of a healthy child, after an easy labour. For the last two years and a half she has been subject to constant and profuse leucorrhœal discharge, with frequent and shooting pains through the region of the uterus, and about the right groin, with occasional dysuria and tenesmus. The general health is greatly disturbed; bowels irregular, with loss of appetite. Upon making an examination per vaginam, pressure of the finger upon the cervix uteri occasioned considerable pain, which, in subsequent examinations, often continued several minutes after the finger had been withdrawn. The os uteri was not indurated, but considerably more open than natural. She had been under the care of several respectable practitioners, and the impression on her mind was that she was labouring under cancer of the womb.

In the first instance the usual mode of treatment was adopted: blood was abstracted by means of cupping from over the inferior portion of the sacrum, to the amount of eight ounces, and repeated three times, with an interval between each of about three weeks. She had taken aperients frequently, and injections of various kinds had been used with little or no benefit.

July 2d.—The nitrate of silver was conveyed by means of the tube, and applied to the cervix uteri for the space of a minute, which occasioned no degree of pain, except what might have been produced by the introduction of the finger.

6th.—The nitrate of silver again applied as before.

9th.—The discharge has diminished, but the pains not having abated, eight leeches were ordered to be applied to the right groin.

July 12th.—The nitrate of silver again applied.

18th.—The discharge is lessened considerably; and the patient now expresses a belief that she shall soon be restored to health, having previously imagined her case to be hopeless.—The nitrate of silver again applied.

27th.—The pain is relieved; general health is improved, and she sleeps well at night. The nitrate of silver applied in the usual manner. It is necessary to observe, that she has taken the hyoscyamus at night, (one drachm of the tincture,) and the bowels have been regulated by aperients. The following tonic has been prescribed: ℞. Infus. Rosæ ℥viiss.; Sulph. Quininæ ʒi.; Elix. Vitriol. ʒi. M. fiat mist. sumantur cochlearia duo ampla ter in die.

August 8th.—The discharge is scarcely perceivable. The nitrate of silver applied as before.

25th.—The patient is perfectly well, having neither vaginal discharge nor local pains.

II. A poor woman, residing in Gardener's row, Westminster, about forty years of age, having several times aborted, had been subject to excessive vaginal discharge for the last eighteen months, with shooting pains through the pelvic region and about the coccyx, and excessive itching of the pudendum. The digestive function was greatly disturbed, and the system exhibited evident proofs of a highly disordered state of the general health. She had taken for a long period different preparations of bark, steel, &c., and had used various injections, with little or no benefit. Blood had also been abstracted locally, by means of leeches. Upon making an examination per vaginam, the cervix uteri was found in the usual irritable and painful state, the margin of the os uteri being perfectly free from induration.

June 12th.—The sponge, as before recommended, was introduced, being well saturated with the solution of the nitrate of silver, in the proportion of three grains to the ounce.

16th.—Applied as before.

19th.—The leucorrhœal discharge is thinner, and less in quantity. The patient was directed to introduce the sponge daily in the same manner.

30th.—Has regularly complied with the directions given, and says she is quite well.

August 2d.—Has had no return of the vaginal discharge, and her appearance is much improved. As a matter of course, attention has been paid to the state of the bowels and the general health.

A case of still greater severity has recently fallen under my notice, which resisted for a very long period all the means which had been tried by several eminent practitioners. At length the iodine was administered, under the influence of which, together with the application of the nitrate of silver, the disease gradually yielded, and the patient is now in perfect health.

I cannot conclude this paper without remarking that there is nothing more empirical than to hold up a particular remedy as a specific in the cure of disease, or to expect it invariably to exert its curative influence upon the function or structure of an organ, under all the diversified circumstances of morbid action. Let it not be imagined that I place such implicit confidence upon the nitrate of silver as to expect it to eradicate, as if by magic, all such diseases as those to which I have adverted: at the same time I confidently believe that, if it be judiciously applied where the vaginal discharge has its origin, or is kept up by inflammation of the cervix uteri or vagina, or by the irritable uterus, and when general principles have not been neglected, there is no remedy so likely to afford such immediate and permanent relief.

*24, Sackville street; September* 1829.

